# “Members of the Same Club”: Challenges and Decisions Faced by US IRBs in Identifying and Managing Conflicts of Interest

**DOI:** 10.1371/journal.pone.0022796

**Published:** 2011-07-29

**Authors:** Robert Klitzman

**Affiliations:** Department of Psychiatry, Columbia University, New York, New York, United States of America; Yale University School of Medicine, United States of America

## Abstract

Conflicts of interest (COIs) in research have received increasing attention, but many questions arise about how Institutional Review Boards (IRBs) view and approach these.

**Methods:**

I conducted in-depth interviews of 2 hours each with 46 US IRB chairs, administrators, and members, exploring COI and other issues related to research integrity. I contacted leaders of 60 IRBs (every fourth one among the top 240 institutions by NIH funding), and interviewed IRB leaders from 34 of these institutions (response rate = 55%). Data were analyzed using standard qualitative methods, informed by Grounded Theory.

**Results:**

IRBs confront financial and non-financial COIs of PIs, institutions, and IRBs themselves. IRB members may seek to help, or compete with, principal investigators (PIs). Non-financial COI also often appear to be “indirect financial” conflicts based on gain (or loss) not to oneself, but to one's colleagues or larger institution. IRBs faced challenges identifying and managing these COI, and often felt that they could be more effective. IRBs' management of their own potential COI vary, and conflicted members may observe, participate, and/or vote in discussions. Individual IRB members frequently judge for themselves whether to recuse themselves. Challenges arise in addressing these issues, since institutions and PIs need funding, financial information is considered confidential, and COI can be unconscious.

**Conclusions:**

This study, the first to explore qualitatively how IRBs confront COIs and probe how IRBs confront non-financial COIs, suggests that IRBs face several types of financial and non-financial COIs, involving themselves, PIs, and institutions, and respond varyingly. These data have critical implications for practice and policy. Disclosure of indirect and non-financial COIs to subjects may not be feasible, partly since IRBs, not PIs, are conflicted. Needs exist to consider guidelines and clarifications concerning when and how, in protocol reviews, IRB members should recuse themselves from participating, observing, and/or voting.

## Introduction

Conflicts of interest (COIs) in medical research have recently been receiving increased attention, but many questions arise about how IRBs view, identify, and manage these problems. COIs are “conditions where a “primary interest” (e.g., patients' welfare) is “unduly influenced by a secondary interest (such as financial gain)” [1]. COIs can be both financial and non-financial (e.g., involving desires for professional recognition, etc. [2]), and can bias research [1], [3]–[7].

Attention to COIs increased after Jesse Gelsinger, an 18-year-old “normal,” healthy volunteer, died in a 1999 University of Pennsylvania gene therapy experiment. Subsequently, the principle investigator (PI) was found to hold shares in the company that stood to gain millions of dollars, depending on the results. Since then, COIs among PIs [8]–[10], as well as IRB members [11] have been increasingly documented. Generally, PIs must now disclose COIs to universities, and can hold no more than $10,000 interest in a company whose products they are studying [12]. The Institute of Medicine (IOM) [13] recommends that consent forms include basic information about COIs and state that additional information is available on request. But whether this approach suffices, how much detail should be provided, who would supply it (e.g., a COI office or IRB), how often subjects request it, and how they understand it, are unclear.

Key questions remain concerning the roles of IRBs in identifying and managing their own and PIs' COIs. Federal regulations prohibit US IRB members with COIs from participating in reviews except “to provide information requested by the IRB” [14]. Yet in one study, though 36% of IRB members had financial relationships with industry, 23% of those never disclosed it to the IRB, and 19.4% nonetheless always voted on the protocol [11]. Of medical center IRBs, one-third do not require that members disclose financial COI [15], yet one-third of IRB chairs do not always arrange for members with COI to leave the room when the protocol is discussed. Others argue that IRB members should disclose COIs, but still participate in IRB deliberations, as they may have relevant expertise [16].

Yet surprisingly, no studies have examined how IRBs view and make these decisions. The vast majority of publications on COI have been theoretical; and the relatively little empirical work conducted in this area has been quantitative, and examined only financial, but not other COIs.

Given the many unexplored questions concerning how IRBs identify and manage PIs' and their own COI, I conducted an in-depth semi-structured interview study of views and approaches among IRB chairs, administrators, and members toward COI and other aspects of research integrity [17]. The study aimed to address several critical gaps in knowledge concerning how IRBs view and approach COI and other integrity issues – e.g., what kinds of financial and non-financial COI issues IRBs confront, how they identify and manage these, what challenges they face in doing so, and how they make and view these decisions. Given the dearth of knowledge about these questions – the fact that no studies have examined how IRBs make decisions about COIs or identify or manage non-financial COI among IRBs – a hypothesis-generating approach is crucial. I thus used qualitative methods to allow for detailed explorations of issues that emerged in order to understand these domains as fully as possible. The interviews shed light on several other issues as well, regarding central IRBs [18], variations between IRBs [19], and research ethics in the developing world [20].

## Methods

As described elsewhere [18], I conducted in-depth interviews of 2 hours each with 46 chairs, directors, administrators, and members. I contacted the leadership of 60 IRBs around the country, representing every fourth one in the list of the top 240 institutions by NIH funding, and interviewed IRB leaders from 34 of these institutions (response rate = 55%). In some cases, I interviewed both a chair and/or director, as well as an administrator from an institution (e.g., as the chair thought that the administrator might be better able to answer certain questions). From these 34 institutions, I thus interviewed a total of 39 chairs/directors and administrators. I also asked half of these leaders (every other one interviewed on the list by amount of NIH funding) to distribute information about the study to members of their IRBs, in order to recruit 1 member of each of these IRBs as well. Thus, I interviewed 39 chairs/directors and administrators, and 7 other members (1 community, and 6 regular members).

The interview guide (see Appendix S1) sought to obtain detailed descriptions of participants' views of RI, COI, IRB responses, and factors involved. The methods were informed by Grounded Theory [21].

I drafted the questionnaire, drawing on prior research I conducted and published literature. Transcriptions and initial analyses of interviews occurred during the period in which the interviews were being conducted, and these analyses helped shape subsequent interviews. The Columbia University Department of Psychiatry IRB approved the study. All participants gave informed consent.

Once the full set of interviews were completed, subsequent analyses were conducted in two phases, primarily by myself and a trained research assistant (RA).

In phase I, we independently examined a subset of interviews to assess factors that shaped participants' experiences, identifying recurrent themes and issues that were then given codes. We read each interview, systematically coding blocks of text to assign “core” codes or categories (e.g., discussions of COI, or of federal audits of IRBs and institutions). A topic name (or code) was inserted beside each excerpt of the interview. We then worked together to reconcile these independently developed coding schemes into a single scheme, and prepared a coding manual, defining each code and examining areas of disagreement until reaching consensus. We discussed new themes that did not fit into the original coding framework, and modified the manual when appropriate.

In phase II of the analysis, we independently performed content analysis of the data to identify the principal subcategories, and ranges of variation within each of the core codes. The sub-themes identified by each coder were reconciled into a single set of “secondary” codes and an elaborated set of core codes. These codes assess subcategories and other situational and social factors (e.g., different types of COI, and ways IRBs address these).

Codes and sub-codes were then used in analysis of all of the interviews. To ensure coding reliability, two coders analyzed all interviews. Where necessary, multiple codes were used. We examined areas of disagreement through closer analysis until consensus was reached through discussion. We checked regularly for consistency and accuracy in ratings by comparing earlier and later coded excerpts.

To ensure that the coding schemes established for the core codes and secondary codes are both valid (i.e., well grounded in the data and supportable) and reliable (i.e., consistent in meaning), they were systematically developed and well-documented. We used Microsoft Word to manage and analyze the data, and search for words and phrases.

## Results

As summarized in Table 1, the 46 interviewees included 28 chairs/co-chairs; 10 administrators (including 2 directors of compliance offices); and 7 members. In all, 27 were male and 19 were female, and 93.5% were Caucasian. Interviewees were distributed across geographic regions, and institutions by ranking in NIH funding.

**Table 1:** Characteristics of the Sample.

	Total	% (N = 46)
**Type of IRB Staff**		
Chairs/Co-Chairs	28	60.87%
Directors	1	2.17%
Administrators	10	21.74%
Members	7	15.22%
**Gender**		
Male	27	58.70%
Female	19	41.30%
**Institution Rank**		
1–50	13	28.26%
51–100	13	28.26%
101–150	7	15.22%
151–200	1	2.17%
201–250	12	26.09%
**State vs. Private**		
State	19	41.30%
Private	27	58.70%
**Region**		
Northeast	21	45.65%
Midwest	6	13.04%
West	13	28.26%
South	6	13.04%
**Total # of Institutions Represented**	**34**	

**Figure 1:**
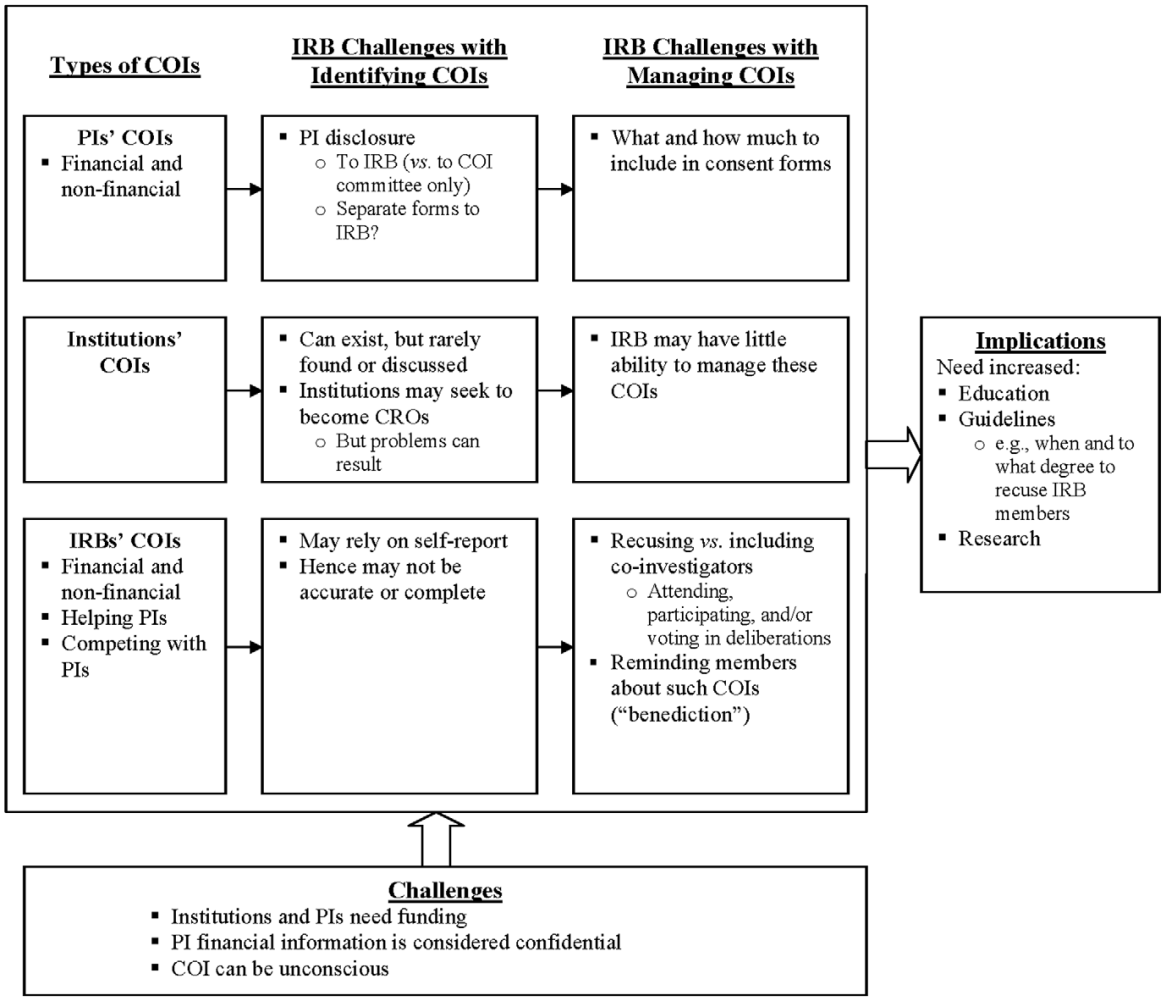
Issues Concerning Identification and Manage of IRBs' Own and Others' COIs.

As outlined in Figure 1, and described more fully below, IRBs confronted several broad categories of financial and non-financial COIs of PIs, institutions, and IRBs themselves. IRB members may seek to either help, or compete with, principal investigators [PIs]). IRBs faced challenges and uncertainties in identifying and managing these COI, and did so in several ways, and felt their responses could potentially be more effective. These issues are categorized below as: IRB's identification and then management of COIs among PIs, institutions, and IRB members. However, these categories overlap – i.e., identification and management issues are often closely intertwined. Financial and non-financial COI are also frequently hard to disentangle.

### Identifying PIs' COIs

IRBs were often uncertain how to define PIs' “COI.” The $10,000 cut-off may not be ideal, since it could mean much more to a junior than a senior PI. Hence, IRBs were also unclear whether research should “not even have “the shadow of an appearance” of a COI.

Financial COI were also hard to identify and assess because the future profitability of an investigational product is uncertain. PIs might accrue profit only in the future, and hence not self-report or perceive a COI.

Often, surgeons don't realize that developing equipment they might eventually sell, or have some interest in, is a conflict. They are not getting money *currently*. **IRB17**

Financial COIs can also be *indirect* – e.g., a PI getting paid through an industry-supported CME course. IRBs debated whether to look “beyond” the immediate source of money alone.

IRBs also face questions of *who* should identify others' COIs, and how engaged to be. Usually, another institutional office addresses many aspects of COIs, but IRBs nevertheless become involved to varying degrees. With institutional COI offices, IRBs had varying relationships that IRBs often felt were inadequate. One IRB only knew of a major PI COI because of sharing an office with the grants department. IRBs may also learn of a PI's COI from an NIH grant, not from the PI.

There's not enough interaction between the COI committee and the IRB. If PIs declare they have a conflict, it goes to the COI committee to manage or eliminate. But the committee usually doesn't send us back a report. They take care of the COI, and send a letter saying, “Yes, we are taking care of it.” But we may not know…how… **IRB17**

The IRB may know only that a plan has been put into place, and many deduce, but not explicitly apprehend, details. “We just get told there's a conflict, and now a plan. It's clear when there's a change of PI. We figure that's how they're resolving the issue” (**IRB3**).

But resolving COIs is often not straightforward, and simply making a PI a co-investigator may not wholly eliminate it.

Hence, some IRBs have developed a second, separate COI form, but then face challenges.

The university's system for annual COI disclosure is only on-line, and not very good. So, the IRB looks on its own at protocols to see if there's any conflict. Every now and then, there is one. **IRB18**

The quality of existing COI forms can also thus range widely.

But IRBs may then require that PIs fill out long, additional COI questionnaires. These instruments may rely, however, on PIs' self-disclosures, which might not be accurate. “We count on the researchers telling us. It's fairly reliable, but not perfect. They might not perceive a conflict that we do” (**IRB17**).

PIs may resist or refuse disclosing COI information to an IRB, which they see as “wanting to know everything.”

Particularly at smaller institutions, confidentiality concerns may arise since individuals reviewing the information may know PIs fairly well, and organizational structures may be less formalized. (“Some things are *too personal*. I wouldn't want to be responsible for keeping that confidential” [**IRB13**]).

PIs may submit differing information on institutional and IRB COI forms – often because conflicts having now been managed. Yet IRBs still have to investigate.

Investigators mark the forms incorrectly 15% of the time. It could be oversight. Or, the PI had managed and gotten rid of the conflict, and just not updated the database. **IRB1**

Questions thus arise of whether these additional forms and processes can be streamlined.

But most IRBs felt too burdened already to adopt added COI responsibilities. (“We are already in charge of so much” [**IRB17**]). IRBs may want the added information, but not the added work.

Questions arise, too, about identifying and managing potential COI in minimal risk research (e.g., structured instruments may be copyrighted, involving financial gains).

Many interviewees thought that PIs' *non-financial* COI were ubiquitous, but elusive, and hence hard to define, identify, and manage. IRB members usually acknowledged that in the academic culture of “publish or perish,” PIs had to accrue some career gain (e.g., publications, promotions, and grants), creating indirect COI that can be difficult to discover and monitor. “Non-financial” COIs may manifest themselves in PIs' aggressiveness in recruiting and enrolling subjects. Scandals (e.g., the Gelsinger case) may result from PI egos, and thus continue: “The researchers were very aggressive, and wanted to recruit, and treat…That's going to happen. Researchers have their own egos and money involved” (**IRB25**).

Yet IRBs tended not to explicitly seek PIs' non-financial COIs.

### Identifying Institutions' COIs

IRBs may also identify departmental or institutional COIs, but feel unable to manage these. IRBs struggled with whether to assess and respond more aggressively to such COIs. Funding could go to the department, not the PI directly, but still constitute a COI. Such conflicts can be less obvious, and hence overlooked, and IRBs may avoid discussing them. “[These] COI issues can be far more subtle. And there aren't any guidelines on that. People are reluctant to open up that box” (**IRB12**).

Institutional COI could also be “non-financial” (i.e., involving reputation), but not be easily identified by IRBs or other offices. The IRB or COI committee reporting to a CEO could itself represent a COI. Similarly, institutional officials might try to block research that may question the institution.

Our institution has very specific guidelines for regulating faculty members' COIs, but not the *institution's* COI – those of the hospital or medical school. The IRB has to regulate those…Health policy faculty wanted to look at the health records of university employees to determine if lower paid employees had the same access to health care as higher paid employees…The university administration and president disallowed the protocol, saying they were afraid about loss of confidentiality of employees' health records. But, in fact, *they have a COI*: what if these researchers find that access to healthcare is not equitable? The university is at risk, and is judging whether the research can be done. That's a COI. In other research, the hospital itself stands to gain, which can affect the review of protocols. **IRB12**

### Identifying IRB Members' COIs

IRBs may not identify their own COIs well. Some IRBs may see more drug company research as desirable, and feel that they may receive more drug company business if they review protocols quicker. Industry-funded PIs may also try to “game” the system, and engage in “IRB shopping.” Some interviewees felt that that such practices did not lower the quality of the review, but others were more wary.

Pharmaceutical companies will throw more research at you if you have a proven track record. So, from a *business perspective*, faster turn-around pays off…As long as you don't sacrifice the quality of review, but turn studies around relatively quickly, everyone benefits – clients, human subjects…and the pharmaceutical companies. They get their approvals quickly, and we get to enroll as many subjects as they need. **IRB9**

IRBs may see themselves as a business, and highlight business rather than only ethical outcomes. “Most PIs have good *business experience* working with us, and are satisfied with the 30–40 day turnaround” (**IRB9**).

Institutions may also set up their own contract research organizations (CROs) with separate IRBs, giving PIs choices of where to submit protocols. Several institutions may co-establish a de facto CRO.

The objective was to bring clinical trials to the host institutions. It…now involves an IRB. It is similar to a CRO…Its focus is *industry*. **IRB9**

This director feels that the CRO IRB has advantages over his own. “They're quicker…we're not as equipped. They have more resources…it's a *one-stop shop*” (**IRB9**).

At times, interviewees perceived COIs among other IRBs – especially for-profit IRBs. As members of local IRBs, almost all interviewees thought these commercial boards had financial COIs. (“They're in it for the money…” [**IRB4**]). Many perceived and feared lower standards as a result.

I'm not really sure what standards these so-called private IRBs uphold, where a doctor doing a study out of their private practice would just pay money and get some IRB to approve it. My impression is that the standards are not as high. I've been involved in many multi-site trials, and someone said, “That design isn't going to fly with the university's IRB, but the private IRBs will accept it.” **IRB12**

IRB members can also have *non-financial* COI, but identify or manage these poorly, if at all. As institutional employees, and often PIs, IRB members may “wear many hats,” and try to help, not impede, colleagues or the institution. One female co-chair said,

We are all part of the same club. When a PI came in to explain his study, it was like a *locker room*. The guys were very friendly, chumming around…The IRB is supposed to be objective, professional. But on a personal level, it may not be as strict or stringent with friends or long-standing colleagues than a regional IRB, with no personal relationships, just dealing with the facts, regulations, and principles…To what extent do the facts that we are all colleagues, and know the investigators, affect our performance – our ability to protect subjects? **IRB40**

The answer to this question is unclear, yet in this regard, centralized IRBs may be more objective.

### Managing PIs' COIs

Generally, IRBs recognize colleagues' and institutions' needs for industry funding, but face challenges. Usually, IRBs try to accept a degree of strain and negotiate, rather than totally eliminate conflicts (except those clearly beyond $10,000).

Many institutions and/or IRBs now require inclusion of PIs' COIs in consent forms, but differ in how and to what degree. COI committees may make these decisions, but IRBs may then have to decide the specific language. IRBs may thus debate how much and what information to include. But IRBs don't know whether participants know how to interpret and evaluate the information, whether these disclosures are sufficient, and/or decrease enrollment, and how to decide.

IRBs may also be more conservative than the institutions' COI committees in having “zero tolerance” for COI, and more power to restrict PIs. “We're more stringent than the med school. We can do whatever we think needs to be done” (**IRB31**).

IRBs struggle with whether and how to manage COIs with relatively low-risk studies, too. One PI, e.g., studied a low-risk nutritional product that still posed COI questions.

He held interest in a company promoting a nutritional product he was studying. It was very low risk, so would probably have had no consequences, but *didn't seem ethically right*. So we proposed that the data be collected and analyzed anonymously. **IRB27**

Yet IRBs tended not to seek to manage PIs' non-financial COIs in any way.

### Managing IRB Members' COI

IRBs face dilemmas, too, of how, and to what degree to manage their own COIs, and do so in several ways. They often try to be “above” finances. (“We try consciously to be *purer* than money. It's important that safety predominates” [**IRB25**]). But that goal may not always be entirely realistic.

IRBs usually seek to manage their own COIs through recusals, but face dilemmas of whether conflicted members can hear, join, and/or vote in deliberations, and how to decide. Chairs may tell members with potential COIs to recuse themselves, but definitions of such COIs (e.g., whether these include non-financial COIs) can be unclear.

At meetings, the chair reminds everybody: if you have a conflict, identify and disclose it, and if you need to, recuse yourself. They then leave the room before the vote. They might stay for *some* of the discussion, and answer questions. **IRB18**

IRBs may bar members from discussions, or leave these decisions to individual members, not all of whom may excuse themselves. IRBs may also suggest that members recuse themselves to avoid pressure from dissatisfied PIs. But these members may continue.

A resident on the IRB reviews protocols, and tells his faculty what he thinks is wrong. We've told him he can recuse himself – that he needs to worry that his department may not like him rattling cages, if he's only a resident…But he feels OK. **IRB32**

The specifics of the department, and people involved, thus differ. Yet permitting members to make these choices on their own can generate problems.

Conversely, members may recuse themselves from reviewing competing researchers' protocols. Yet these abstentions can present tradeoffs, hampering maintenance of a quorum of expertise. Some IRBs consist of “fairly senior people” (**IRB17**), involved in many studies, who may then often have to recuse themselves.

Questions arise, too, of *when* exactly members should recuse themselves – e.g., when *any* protocol from a department is discussed *vs.* those in which they are more closely involved.

Unconflicted experts in a field can be hard to obtain. Chairs may have to push outsiders to review.

We have a limited pool of experts. One cardiologist…consults for all the companies, so can't review for us…But then no one on the committee has the expertise. In some ways, it's better to have someone involved who knows the field. **IRB3**

IRBs may thus face difficult choices.

Some IRBs felt that having a member whose research team's work is being reviewed can be both good and bad.

We've been pretty good about not being too cozy. One member is part of a research group…We were pretty tough on protocols from his group, and he tried to defend or explain what the researcher was doing, and the discussion didn't go his way. On balance, it's been helpful to have him to explain the research, because on the face of it, the research seems a little crazy…exposing healthy subjects to a particular drug. He can explain it… **IRB40**

IRBs may attempt to manage COIs, particularly non-financial ones, in other ways, too. One chair begins every meeting with a “benediction,” suggesting a *ritual* – a systematic means of addressing difficult emotions.

We start every meeting with a *benediction*, saying, “The things that we talk about in this room can affect the careers of the individuals involved. *What goes on in this room stays in this room*. Consider it like Las Vegas: we don't discuss anything outside of these doors.” We really do let our hair down…and call a spade a spade during those meetings. An individual could be doing research for the company that makes a drug, or a competitor, and be conflicted. We allow them to take part in discussion, but not vote. **IRB4**

Chairs thus vary in whether they permit conflicted members to join discussions. Conflicted members in the room, even if not voting, could, by observing colleagues' comments and/or votes, potentially sway decisions.

## Discussion

This study, the first to provide qualitative data on how IRBs confront COIs, and the first to explore how IRBs face non-financial COIs, reveals that US IRBs struggle to identify and manage several types of COIs in various ways. IRBs often wrestle with dilemmas of how to define COI, and balance competing priorities.

While certain aspects of financial COI among IRBs have been probed quantitatively [6], the present study highlights additional ambiguities concerning how IRB members confront non-financial COI – e.g., whether members with non-financial conflicts should leave the room. Members can potentially either aid or hamper PIs, trying to “help their buddies,” or stymie competitors, highlighting both informal and formal interactions between IRB members and PIs. These impediments to reviews can be subtle, subjective, and invisible, and thus hard to detect, manage, or prevent. IRBs' responses to their own potential COI range considerably, and in protocol deliberations, conflicted members may observe, participate, and/or vote. Mechanisms for handling these COI often appear informal, handled case-by-case, *ad hoc*.

Definitions of COI can be blurry (e.g., if PIs are developing a device they might sell in the future). IRBs struggle with whether they should use, as a standard, PIs not having even the *appearance* of a COI, and whether and how much to address COI in minimal risk studies.

These data highlight critical questions concerning definitions, identifications and management of *non-financial* COIs, too. While prior discussions distinguish between financial and non-financial COI, distinctions arise here more commonly between *direct* and *indirect* financial COIs. Non-financial COI have been described, based on political or other commitments (e.g., being a smoker) [22], allegiance to a particular theoretical framework [23], or career advancement and ambition [2], [24]. But the present data reveal “indirect financial COIs” based on gain or loss not to oneself, but to one's colleagues or larger institution.

At times, IRBs themselves wrestle with competing priorities – e.g., managing COI *vs.* having a quorum, and/or sufficient expertise; and avoiding COI *vs.* helping the institution. To gauge COIs, IRBs often rely on trust of PIs and each other – e.g., “knowing” that local PIs would not have a COI, and/or would readily report it. But such trust may not always be fully justified. Physicians may be unconscious of COIs [25], and deny these; and so, too, may many IRB members. Similarly, IRBs may under-appreciate how COIs may affect them, feeling immune, and hence minimizing or denying these. These issues need to be examined far more closely by IRBs, policy makers, and others, and raise broader dilemmas of how to assess trust.

One could argue that IRB COIs with PIs can cut both ways – helping or hindering PIs – and that these phenomena in effect thus cancel each other out. But such “balancing” may not always occur. Rather, at any one time, an IRB may tilt more one way or the other.

These data highlight inefficiencies in current bureaucratic structures for overseeing COIs. Separate institutional COI offices, other than IRBs, may know of, and manage PIs' financial COIs. But IRBs may not then learn of the resultant decisions, though IRBs would often like to, in order to review protocols fully, and assess consent forms. Thus, these institutional structures and relationships can be improved, to share relevant information.

Disclosures of both financial and non-financial COIs to study subjects, though suggested [26], may not be feasible or realistic for indirect financial COI. Partly, IRBs, not PIs, are conflicted. It may not make sense for informed consent forms to state, essentially, “The IRB may also have tried to help the PI by approving this study.” In academic institutions, needs for career advancement pose intrinsic, indirect, and non-financial COI. Consequently, enhancing awareness and education about these COIs among IRBs, chairs, members, and administrators, PIs, and other institutional officials is critical.

Needs exist to consider possible guidelines and clarifications concerning when and how IRB members should recuse themselves from discussing, reviewing, and/or voting on, protocols. IRB members may know many PIs, potentially posing COIs. Hence, the criteria should not be whether members know the PI, but to what degree that knowledge may affect deliberations. Yet these determinations can be highly subjective. Nevertheless, guidance can potentially help members, chairs, and administrators make these decisions.

Central IRBs, including for-profit IRBs, though increasingly proposed [27], may also have inherent COIs. For-profit IRBs get paid to approve protocols [7], yet interviewees may also have COI in potentially wanting to aid or hinder PIs. Still, debates about CIRBs rarely mention that local IRBs may have certain COIs, too. Both local and centralized IRBs may face potential COIs that need to be weighed in discussions about changing the current system.

Additional research is needed, too, to assess more fully when, how, and in what ways direct and indirect financial COIs in fact affect IRBs, and how and to what degree educational or other interventions can help. Such research will face challenges, but is important in optimally protecting participants.

This study has several potential limitations. These data are based on interviews with individual IRB chairs and members, but not direct observation of IRB decision-making, or collection of written IRB records. Future research can also observe IRBs and examine such records. But these data may be difficult to obtain if IRBs require that all the IRB members, PIs, and funders involved provide informed consent. Nevertheless, the present data provide important insights on these issues. These interviews also probed respondents' experiences and views in the present and recent past, but not prospectively. IRBs and Research Ethics Committees (RECs) abroad may also vary, and can be explored future research.

Nevertheless, this study highlights how US IRBs confront several types of COI, and face challenges and ambiguities in defining, identifying, and managing these tensions. Further research and attention concerning these issues is urgently needed.

## Supporting Information

Appendix S1 Sample Questions from Semi-Structured Interview.
(DOC)
