# Supplementary material for: “Members of the Same Club”: Challenges and Decisions Faced by US IRBs in Identifying and Managing Conflicts of Interest
Source: PLoS One. 2011 Jul 29;6(7):e22796. doi: 10.1371/journal.pone.0022796 (PMC3146515; doi:10.1371/journal.pone.0022796)
Supplement: Appendix S1 — Sample Questions from Semi-Structured Interview. (DOC) [file pone.0022796.s001.doc]

**Appendix S1. Sample Questions from Semi-Structured Interview***

- How do you define integrity in research? Has your IRB faced issues concerning COI? If so, where and how? What issues were involved? What did you decide? Why? What have been the most difficult cases concerning COI and RI that you have faced? Do you think IRBs and PIs view COI differently or apply standards differently, and if so, how?
- What are the barriers and facilitators in IRBs monitoring and addressing COI and other RI issues? Do you perceive any gray areas or problems weighing issues about COI? If so, what?
- What do you think makes an IRB work well or not in monitoring and responding to COI?
- Do you have any other thoughts about these issues?

**Note: Additional follow-up questions were asked, as appropriate, with each participant.*
